# Genomic variance partitioning of carcass and meat quality traits in Angus beef cattle

**DOI:** 10.3389/fvets.2025.1590226

**Published:** 2025-06-18

**Authors:** Hasan Baneh, Nikolay Elatkin, Laurent Gentzbittel

**Affiliations:** ^1^Project Center for Agro Technologies, Skolkovo Institute of Science and Technology, Moscow, Russia; ^2^Animal Science Research Department, Kurdistan Agricultural and Natural Resources Research and Education Center, Agricultural Research Education and Extension Organization (AREEO), Sanandaj, Iran; ^3^LLC “Miratorg-Genetika”, Moscow, Russia

**Keywords:** variance partitioning, genomic relationships, genetic architecture, carcass traits, Angus beef cattle

## Abstract

This study aimed to partition the genomic variance of carcass weight (CW), marbling score (MS), rib-eye area (REA), and back fat thickness (BFT) traits in Angus beef cattle into components associated with minor allele frequency (MAF) bins, functional annotation classes, and chromosomes. The dataset included 6,511,978 (6.5 million) imputed whole-genome sequence (WGS) SNPs from 13,241 Angus beef cattle. Genomic partitioning was performed using a multi-component mixed linear model analysis, modeling random effects with multiple genomic relationship matrices (GRMs), either simultaneously (joint analysis) or separately (separate analysis). The estimated heritability (*h*^2^) for CW, MS, REA, and BFT, obtained by fitting all 6.5 million SNPs at once, was 0.22 ± 0.01, 0.25 ± 0.01, 0.35 ± 0.01, and 0.15 ± 0.01, respectively. The aggregate genetic variance components estimated from the separate analysis were substantially larger than the corresponding heritability estimates, while the results of joint analysis for all partitioning factor were very close to *h*^2^ estimates for all traits. A weak relationship was observed between chromosome length and its heritability (*R*^2^ < 0.35). Although intergenic and intronic variants significantly contributed to the genetic variation of the traits, the variance captured per SNP was considerably lower for these variants compared to genic variants, particularly exon variants.

## 1 Introduction

Carcass weight and meat quality are economically important quantitative traits in the global beef industry. These traits are influenced by both environmental factors and the genetic effects of numerous loci distributed across the genome (1). The advent of advanced genotyping technologies has provided new opportunities to explore the genetic basis of these traits more comprehensively and accelerate breeding programs. In genomic evaluation programs, all available genetic marker genotypes, regardless of the magnitude or statistical significance of their effects, are used simultaneously to predict an individual's genetic merit and estimate variance components (2, 3). For instance, Jensen et al. (4) and Román-Ponce et al. (5) reported that a substantial proportion of the additive genetic variance for production and fitness-related traits in dairy cattle can be captured using genomic information from medium-density SNP panels (e.g., the Bovine 50K SNP array). While genomic information has improved the prediction accuracy of selection candidates, it is often applied as a “black-box” prediction due to limited understanding of the genetic architecture of traits (6). Daetwyler et al. (7) demonstrated that the accuracy of genetic and genomic predictions for complex traits is strongly influenced by their underlying genetic architecture. Therefore, gaining a deeper understanding of this architecture has the potential to further enhance the accuracy of predictions in livestock breeding programs.

The ENCODE project revealed that only 1.22% of the human genome encodes defined products, while ~80% is involved in biochemical activities (8), highlighting the functional importance of nearly the entire genome. However, the contribution of different functional annotations to the additive genetic variance of complex traits remains controversial. Published studies suggest that a larger proportion of genetic variance in humans (9, 10), dairy and beef cattle (2), and broiler chickens (11) can be attributed to genic regions. In contrast, other studies have reported that intergenic and intronic regions explain a higher proportion of genetic variation in beef cattle (12). Similarly, Do et al. (13) found that genic and non-genic regions contribute approximately equally to the variation in feed efficiency traits in pigs. Additionally, Morota et al. (14) observed that the contribution of different genomic regions to genetic variation varies across traits. These findings highlight the importance of dissecting genomic variance to improve the predictive performance of genetic evaluation models.

The allele frequencies of various genomic regions change dynamically over generations due to factors such as artificial selection in livestock species. In genomic studies, markers with low minor allele frequencies (MAF; e.g., < 0.01 or < 0.05) are often discarded during quality control (QC) steps. However, these markers may contribute to complex traits (15). Discarding these SNPs could result in significant information loss and hinder the detection of rare disease-associated markers (16). Interestingly, SNPs with low MAF (< 0.1) have been shown to be more effective in detecting quantitative trait loci (QTL) with low MAF compared to SNPs with higher MAF (> 0.4) (11). Several studies (1, 3, 17) have reported substantial variation among MAF bins in their contribution to the variance of complex traits in beef and dairy cattle breeds. Furthermore, estimating the genetic variance explained by individual chromosomes can provide insights into the genetic architecture of traits. A strong correlation between chromosome heritability and physical length is expected if a trait is influenced by many loci evenly distributed across the genome (11, 18). However, a weak relationship has been reported for meat quantity and quality traits in Hanwoo cattle, suggesting that major genes are not evenly distributed across the genome of this breed (1).

Partitioning the genetic variance of complex traits across different subject categories, such as functional annotations, can enhance our understanding of the genetic architecture and inheritance mechanisms underlying these traits. Although SNP chip arrays offer several advantages, they capture only a portion of genetic variation due to incomplete linkage disequilibrium (LD) with causal variants (19–21). For instance, while the cattle genome is reported to contain ~26.7 million variants (22), SNP arrays typically cover only a small subset of these variants. Koufariotis et al. (2) have advocated for the use of whole-genome sequence (WGS) data to address this limitation, since it offers more comprehensive genomic coverage than SNP arrays.

Genotype imputation is a promising approach for increasing the marker density and enhancing LD between SNPs and causal variants. It also improves the representation of rare variants and the availability of causal variants themselves (3, 21, 23). To the best of our knowledge, the genomic partitioning of carcass weight and meat quality traits has not yet been studied in Angus beef cattle. Therefore, this study aimed to utilize imputed SNP data at the whole genome sequence level to investigate the genetic variance of carcass weight, marbling score, back fat thickness, and rib-eye area in Angus beef cattle.

## 2 Materials and methods

### 2.1 Population and herd management

The data were collected from 50 Angus beef cattle farms, with the number of samples per farm ranging from 57 to 1,706 and an average of 264 samples per farm. The farms are geographically close, experience comparable climate conditions, and are genetically connected. All farms operated under the same management system. Newborn calves were kept with their mothers till weaning (4–6 months) and then were transferred to pastures, where they were fed until reaching 12 to 15 months of age and ~350 kg in weight. The calves were then moved to feedlots, where they were raised for about 7 months.

### 2.2 Phenotypes

The studied traits included carcass weight (CW, kg), marbling score (MS, score), rib-eye area (REA, in^2^), and back fat thickness (BFT, mm). Phenotypic records were collected from 13,241 steers born between 2017 and 2019. These animals were slaughtered at an average age of ~700 days. Marbling score was measured between the 12^th^ and 13^th^ ribs using a special automatic scanner that grades meat on a scale of 1 to 12. The rib-eye area was recorded as the total area of the loin (*longissimus dorsi* muscle). Back fat thickness represented the external fat on the carcass, measured between the 12^th^ and 13^th^ ribs. All four traits were measured in all individuals. Descriptive statistics of the studied traits are presented in Table 1. Least squares analysis of variance was performed to identify significant environmental effects on the traits. The effects included birth year, birth month, birth farm, feedlot, recording year, recording month, and recording age. All environmental factors were found to be significant and were included as fixed effects in the final model.

**Table 1:** Descriptive statistics and estimated heritability of the traits.

**Trait**	**No. records**	**Min**	**Max**	**Mean**	**SD**	**CV**	**h^2^**
CW (kg)	13,241	288.8	721.1	404.06	37.76	9.35	0.22 ± 0.01
MS (score)	13,241	2.68	10.24	7.26	1.35	18.60	0.25 ± 0.01
REA (in^2^)	13,241	6.91	16.65	11.87	1.33	11.20	0.35 ± 0.01
BFT (mm)	13,241	0.064	1.976	0.731	0.26	35.57	0.15 ± 0.01

CW, carcass weight; REA, rib-eye area; MS, marbling score; BFT, back fat thickness.

### 2.3 Genotypes and imputation

The animals (*n* = 13,241) were genotyped using the Illumina Bovine 50K SNP panel. All samples had a call rate >0.90 and were thus retained for analysis. Duplicate markers, indels, deletions, multiallelic sites, unmapped SNPs, and those located on mitochondrial or sex chromosomes were removed from the dataset. Additionally, SNPs with MAF < 0.05 and SNP call rate < 0.95 were discarded. Following these quality control steps, 39,580 autosomal SNPs were retained for downstream analysis. The quality control process was performed using PLINK v1.07 software (24).

The whole genome sequences (WGS) of purebred Angus cattle were downloaded from the “1,000 Bull Genomes Project” (22). After filtering the sequences for bi-allelic loci, 13,123,690 SNPs were retained. To determine optimum values for quality control metrics of reference sequences and calibrate the input parameters of the genotype imputation program, we performed a pilot study using 5,000 randomly selected SNPs from the target population. The values yielding the highest percentage of correctly imputed genotypes were selected for subsequent genotype imputation. Therefore, the reference dataset was filtered for variants with MAF > 0.02, sequence depth > 3, sequence quality > 30, and a missing rate < 20%. Finally, a total of 9,268,297 SNPs of 128 Angus samples were retained and used as the reference population for the genotype imputation. Genotype imputation was performed using the population-based imputation algorithm implemented in Beagle v4.1 (25). The imputed genotypes were filtered based on dosage R-squared (*R*^2^) > 0.8 and MAF > 0.01. After quality control, 6,511,978 SNPs remained for downstream analysis. More details about the genotype imputation process were reported in our previous work (26).

### 2.4 Variant annotation

The functional annotation of the imputed WGS variants was performed using the Variant Effect Predictor (VEP) online web interface (27) of the Ensembl server (release 106), and the annotations were mapped to the bovine genome assembly ARS-UCD1.2. The functional annotations described in the Ensembl had been classified into 16 categories, with considerable variation in the number of SNPs, ranging from 16 SNPs for stop-retained variants to 3,840,411 SNPs for intergenic region variants (Supplementary Table S1). Therefore, the function annotations were classified into four groups: intergenic region, intron, regulatory region (including downstream and upstream), and exon variants (missense, synonymous, 3′ UTR, 5′ UTR, and other regulatory variants). The distribution of variants across these groups is presented in Supplementary Table S1. The “other regulatory variants” category encompassed splice_acceptor, splice_donor, splice_region, start_lost, stop_lost, and stop_retained variants.

### 2.5 Genomic variance partitioning

The total genetic variance captured by the whole genome was partitioned based on MAF, chromosomal position, and functional annotation of the markers. To investigate the additive genetic variance of traits due to MAF bins, imputed WGS were classified into five bins: 0 < MAF ≤ 0.09, 0.09 < MAF ≤ 0.18, 0.18 < MAF ≤ 0.27, 0.27 < MAF ≤ 0.38, and 0.38 < MAF ≤ 0.5. The bin thresholds were set to ensure each MAF class contained an equal number of SNPs. The markers were initially classified into four main groups to estimate the proportion of genetic variance explained by functional annotations: intergenic, intron, regulatory regions, and exon. However, due to an imbalance in the number of SNPs within these categories, further analysis was conducted to compute the variance captured by each subcategory within the exon group, as described in section 2.6. The contribution of autosomal chromosomes (*n* = 29) to the genetic variance of the traits under study was also investigated.

Two different strategies were applied to partition the total additive genetic variance attributed to each studied factor (MAF, chromosome, and functional annotation). In the first strategy (separate analysis), each category of MAF (*n* = 5 bins), chromosome (*n* = 29), and functional annotations (*n* = 4 groups) was considered separately in a single random effect model. To do this, the genomic relationship matrix (GRM) constructed using the SNPs of the respective category was applied to model the covariance among individuals in a univariate mixed linear model. The statistical model, in matrix form, was as follows:


$$\begin{array}{l} \text{y}=\text{Xb}+\text{Zg}+\text{e} \end{array}$$

In the second strategy (joint analysis), all respective categories of each factor (MAF, chromosome, and functional annotation) were simultaneously fitted in a univariate mixed linear model with multiple random effects. The model was as follows:


$$\begin{array}{l} \text{y}=\text{Xb}+\overset{n}{\underset{i=1}{\sum }}\text{Z}{\text{g}}_{\text{i}}+\text{e}, \end{array}$$

where **y** is the vector of phenotypes; **b** is the vector of fixed effects including birth year, birth month, recording year, recording month, birth farm, feedlots and slaughtering age (as covariate); **g** ~ N(0, **G**$\sigma_{a}^{2}$) is the vector of random additive genetic effects attributed to the i^th^ SNP subset, and **e**~ N(0, **I**$\sigma_{e}^{2}$) is the vector of random residual errors. **X** and **Z** are the incidence matrices relating **b** and **g** effects to **y**, respectively. **G** and **I** are the genomic relationship matrix (defined below) and identity matrix, respectively. $\sigma_{g}^{2}$ and $\sigma_{e}^{2}$ are the genetic variance explained by genome-wide SNPs and the residual variance, respectively. Also, n is the number of subsets for non-overlapping SNP partitions (*n* = 5 for MAF bins, *n* = 29 for the autosomes, and *n* = 4 for the functional annotations). In joint analysis, no covariance among the random effects was assumed. GRM was constructed following the method defined in Yang et al. (28).


$$\begin{array}{l} G_{jk}=\frac{1}{M}\overset{M}{\underset{i=1}{\sum }}\frac{\left(x_{ij}-{2p}_{i}\right)\left(x_{ik}-{2p}_{i}\right)}{2p_{i}\left(1-p_{i}\right)}, \end{array}$$

where ***G***_*jk*_ is the off-diagonal element for animals j and k, or the diagonal element if j = k. Genotype codes of x_ij_ = 0, 1, 2 for A1A1, A1A2, and A2A2, respectively. p_i_ is the allele frequency of A2 at locus i calculated based on population SNP genotype data, and M is the number of SNPs used for GRM construction. To explain the strategies, as an example, we fitted 29 different mixed linear models, each corresponding to a chromosome being analyzed separately (Strategy 1; separate analysis). Additionally, a model including 29 different random effects, each structured by one chromosome, was fitted (Strategy 2; joint analysis). All genetic variance partitioning analyses were conducted using genomic-relatedness-based restricted maximum-likelihood (GREML) estimation implemented in the Genome-wide Complex Trait Analysis (GCTA) program version 1.94.1 (28).

### 2.6 Genetic variance per SNP

Although fitting a multiple-factor model at once (joint analysis) can partition the total genetic variance appropriately and prevent confounding signals from different effects, the estimates could be affected by unbalanced distribution (unequal number of) variants in the fitted effects. In this study, there were notable differences among functional annotation classes regarding SNP numbers. Hence, to gain insight into the genetic variation of the traits explained by each annotation class and to address this issue, the proportion of genetic variance captured by each SNP (VarPerSNP) was estimated. This approach, proposed by Koufariotis et al. (2), is calculated using the following formula:


$$\begin{array}{l} {\text{VarPerSNP}}_{i}=\frac{\left[\left(\frac{h_{i}^{2}}{n_{i}}\right)\;*\;100\right]}{10^{-\;4}}, \end{array}$$

where *h*^2^ is the estimated heritability, and *n* is the total number of SNPs in the i^th^ annotation class. The calculated value for each class was multiplied by 100 to express it as a percentage and divided by 10^−4^ to scale the value for better visualization.

## 3 Results

In the current study, the imputed genotypes at the whole genome sequence level were used to partition the genomic variance of carcass weight and meat quality traits in Angus beef cattle. Genotype imputation significantly increased the information available (>164 times; 39,580 vs. 6,511,978 autosomal SNPs). The distribution of markers across the genome is summarized in Table 2. The average marker interval for the imputed WGS was ~381 base pairs (bp). The autosome marker density showed a consistent pattern, ranging from 316 bp (BTA23) to 442 bp (BTA5). There was a strong linear relationship between chromosome length (the difference of physical position of the last and the first SNP on each chromosome) and number of SNPs (*R*^2^ > 0.93; Supplementary Figure S1). The heritability of the traits was estimated using mixed linear models that accounted for the genomic relatedness among individuals, modeled by the genomic relationship matrix constructed using imputed WGS SNPs. The heritability estimates were 0.22, 0.25, 0.35, and 0.15 for CW, MS, REA, and BFT, respectively.

**Table 2:** Chromosome length, number of SNPs, and summary of marker interval for each chromosome.

BTA^*^	**Length (Mbp)**	**No. SNPs**	**Marker interval (bp)**		
			**Min**	**Max**	**Mean**
1	157.90	451,494	1	240,215	349.73
2	134.37	352,103	1	354,137	381.62
3	120.97	312,673	1	326,021	386.88
4	119.74	312,754	1	286,441	382.86
5	120.04	271,721	1	590,038	441.78
6	117.79	368,198	1	440,976	319.92
7	110.60	282,397	1	290,617	391.64
8	113.10	276,607	1	444,895	408.87
9	104.35	246,313	1	305,590	423.64
10	103.27	273,550	1	1,181,758	377.52
11	106.40	268,698	1	217,101	395.98
12	87.12	229,000	1	982,989	380.42
13	83.26	195,198	1	646,635	426.53
14	82.13	214,646	1	261,799	382.65
15	84.76	215,280	1	365,559	393.70
16	80.94	200,102	1	454,530	404.50
17	73.11	222,995	1	172,473	327.88
18	65.28	149,284	1	233,339	437.28
19	63.40	146,122	1	280,917	433.89
20	71.61	200,773	1	278,968	356.68
21	69.39	159,833	1	654,446	434.17
22	60.58	152,216	1	279,710	398.01
23	52.46	166,250	1	142,342	315.54
24	62.24	172,528	1	136,712	360.77
25	42.31	125,939	1	68,926	335.94
26	51.96	134,892	1	253,127	385.18
27	45.59	130,372	1	133,281	349.67
28	45.88	132,345	1	460,326	346.69
29	51.02	147,695	1	412,660	345.46
Overall	2,481.56	6,511,978	1	1,181,758	381.08

* BTA, Bos taurus autosomes.

### 3.1 Genomic variance partitioning by individual chromosomes

The estimation of genetic variance due to the autosomes was performed using mixed linear models, fitting each chromosome as a random effect. To do this, the covariance among individuals due to each chromosome was modeled using a genomic relationship matrix constructed by SNP genotypes located on that chromosome. Each chromosome was fitted as a random effect in either a multi-factor model (joint analysis) or a single-effect model (separate analysis). The results showed that the total genetic variance of the traits, by summing up the estimates of all autosomes obtained by separate analysis, was 378.51, 0.66, 1.65, and 0.015 for CW, MS, REA, and BFT, respectively. These values were higher than the corresponding estimates obtained from the joint analysis (233.42, 0.413, 0.565, and 0.009, respectively) and those derived from the model fitting the entire set of imputed SNPs (6.5 million) at once (229.58, 0.419, 0.554, and 0.009, respectively).

**Figure 1:**
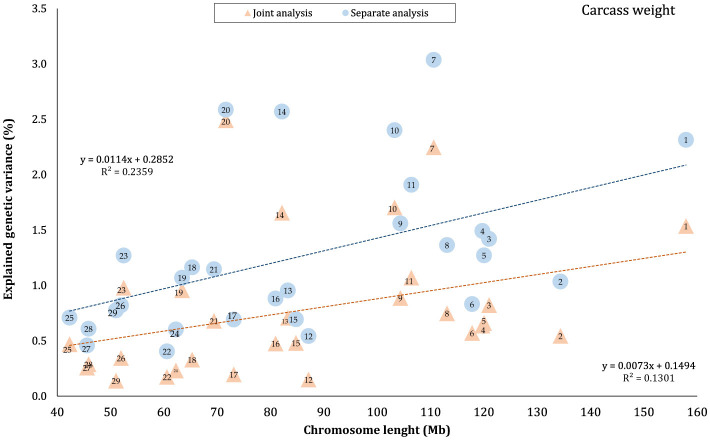
Estimated proportion of variance explained by each chromosome for carcass weight (CW) against physical length of the chromosomes using separate and joint analysis.

**Figure 2:**
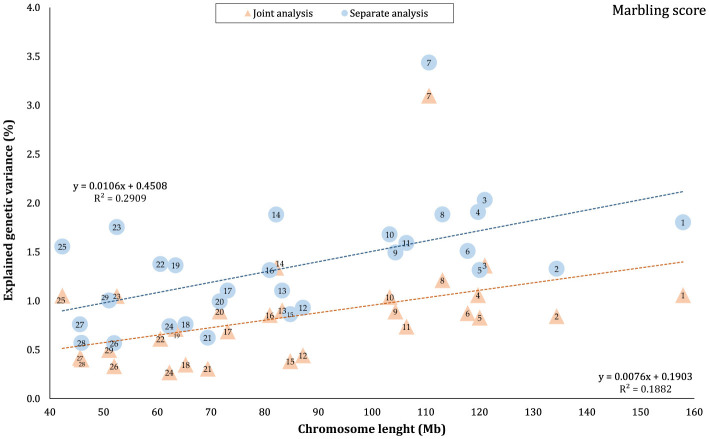
Estimated proportion of variance explained by each chromosomes for marbling score (MS) against physical length of the chromosome using separate and joint analysis.

**Figure 3:**
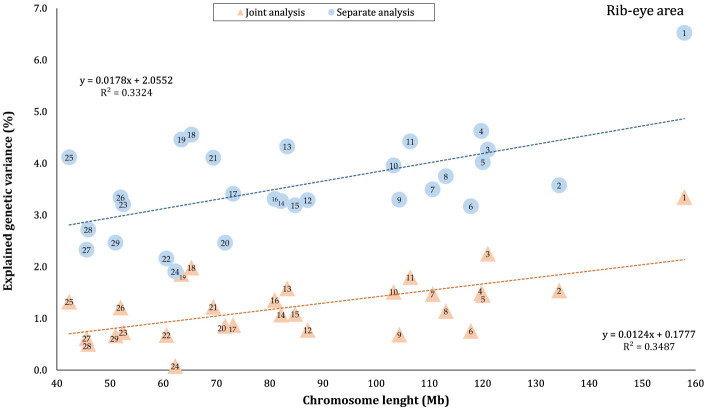
Estimated proportion of variance explained by each chromosomes for rib-eye area (REA) against physical length of the chromosome using separate and joint analysis.

**Figure 4:**
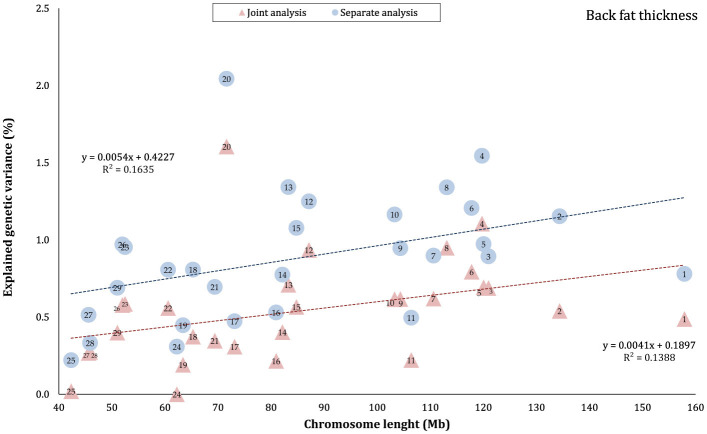
Estimated proportion of variance explained by each chromosomes for back fat thickness (BFT) against physical length of the chromosome using separate and joint analysis.

The proportion of genetic variance of CW, MS, REA, and BFT explained by each chromosome using separate and joint analysis is shown in Figures 1–4, respectively. The estimates are expressed as the ratio of additive genetic variance captured by each chromosome to total phenotypic variance, which is referred to as chromosome heritability. In both joint and separate analyses, the autosomal chromosomes showed a wide range of contributions to the phenotypic variation of the studied traits. For joint analysis, the ranges of chromosome heritability were 0.14% (BTA29) to 2.49% (BTA20) for CW, 0.27% (BTA24) to 3.1% (BTA7) for MS, 0.08% (BTA24) to 3.35% (BTA1) for REA, and 0.0001% (BTA24) to 1.61% (BTA20) for BFT. In the separate analysis, chromosome heritability estimates ranged from 0.40% (BTA22) to 3.04% (BTA7) for CW, 0.57% (BTA26) to 3.44% (BTA7) for MS, 1.91% (BTA24) to 6.53% (BTA1) for REA, and 0.22% (BTA25) to 2.04% (BTA20) for BFT traits. The estimates for joint analysis were generally lower than those for separate analysis, particularly for REA. Additionally, while the chromosome length is highly correlated with the number of variants located on it (Supplementary Figure S1), the relationship between heritability and chromosome length was not as strong (*R*^2^ < 0.35).

### 3.2 Genomic variance partitioning by SNP MAF

**Figure 5:**
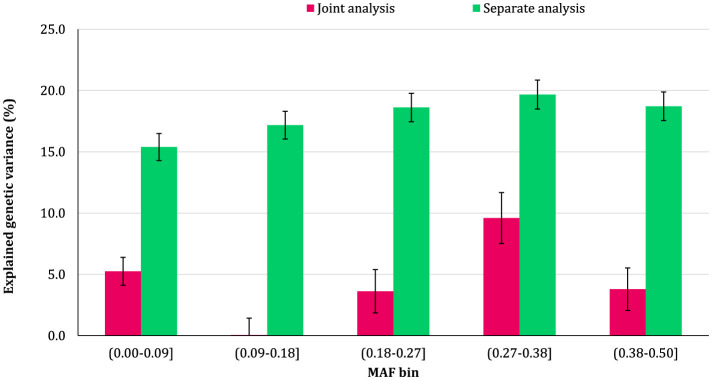
Estimated proportion of variance explained by MAF bins for carcass weight (CW) using separate and joint analysis.

**Figure 6:**
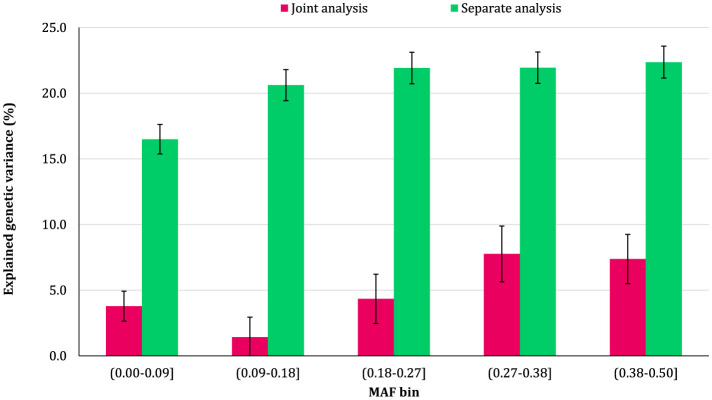
Estimated proportion of variance explained by MAF bins for marbling score (MS) using separate and joint analysis.

**Figure 7:**
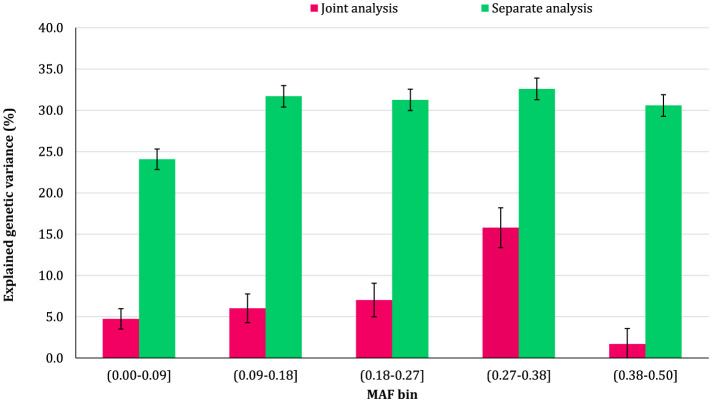
Estimated proportion of variance explained by MAF bins for rib-eye area (REA) using separate and joint analysis.

**Figure 8:**
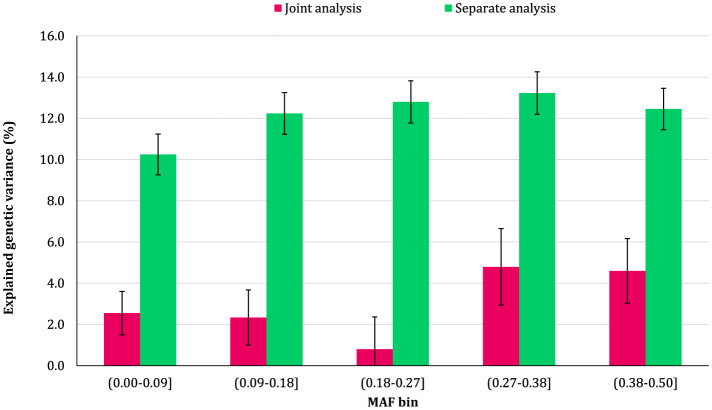
Estimated proportion of variance explained by MAF bins for back fat thickness (BFT) using separate and joint analysis.

The proportion of additive genetic variance explained by SNPs in different MAF bins for the traits is shown in Figures 5–8, respectively. The variance explained by each MAF bin, estimated by separate analysis, was larger than the corresponding values obtained from joint analysis for all traits. In addition, the results revealed that the estimates from fitting five MAF bins separately were within a small range of 15.39–19.67% for CW, 16.49–22.37% for MS, 24.08–32.59% for REA, and 10.25–13.23% for BFT. In contrast, the estimates obtained from the joint model exhibited a wider range: 0.05–9.6% for CW, 1.42–7.76% for MS, 1.71–15.79% for REA, and 0.79–4.79% for BFT.

The total genetic variance captured by all MAF bins (sum of the estimates) from the separate analysis was substantially greater than that captured by all imputed variants modeled as a single effect. For MS and REA, the accumulated variance even exceeded the phenotypic variance of the trait. Conversely, the ratio of the sum of variance components obtained from joint analysis to phenotypic variance was very close to the heritability of the traits. The traits did not exhibit a consistent pattern regarding the proportion of genetic variance explained by different allele frequency classes. In the joint analysis, the highest proportion of genetic variance for all traits was explained by SNPs with 0.27 < MAF ≤ 0.38 bin. This bin accounted for 9.6%, 7.76%, 4.79%, and 15.79% of the phenotypic variance of CW, MS, BFT, and REA, respectively. Interestingly, rare variants explained 5.25% (23.53%), 3.75% (15.33%), 4.75% (13.45%), and 2.55% (16.90%) of the phenotypic (genetic) variance of CW, MS, REA, and BFT, respectively.

### 3.3 Genomic variance partitioning by functional annotation

The whole-genome imputed SNPs were functionally annotated into 15 different groups, as shown in Supplementary Table S1. Due to a very low number of SNPs in some categories, we grouped the SNPs into 4 major classes, including intergenic (58.97%), intron (31.52%), regulatory region (8.66%), and exon variants (0.85%). The results of separate (fitting each annotation class at the time) and joint (fitting all classes together in a multi-random effect model) analysis for the aforementioned annotation classes are summarized in Table 3. The results indicated that when the annotation classes were analyzed separately, each class accounted for a significant proportion of the total genetic variance, much higher than the corresponding values obtained from the joint analysis. The total genetic variances (sum of the estimates) explained by classes in separate analyses were 79.68%, 87.54%, 129.44%, and 53.31% for CW, MS, REA, and BFT, respectively. These estimates were considerably higher than those obtained when all imputed SNPs (6.5 million) were fitted simultaneously. The proportion of total genetic variances due to annotation classes in the joint analysis, relative to phenotype variance, was 21.85%, 24.7%, 35.47%, and 15.62%, respectively, for CW, MS, REA, and BFT. These values were consistent with the heritability estimates obtained using all imputed SNPs.

**Table 3:** Partitioning genetic variance of the studied traits due to functional annotation of genome regions.

**Category**	**No. SNPs**	**%**	**Analysis**	**CW**	**MS**	**REA**	**BFT**
Intergenic	3,840,411	58.98	Separate	20.23 ± 1.2	23.65 ± 1.22	33.09 ± 1.32	13.9 ± 1.07
			Joint	6.18 ± 1.51	11.28 ± 1.67	9.7 ± 1.7	5.97 ± 1.44
Intron	2,052,343	31.52	Separate	20.41 ± 1.17	22.29 ± 1.19	32.26 ± 1.27	13.85 ± 1.05
			Joint	7.78 ± 1.98	9.54 ± 2.03	7.88 ± 2.14	6.09 ± 1.79
Regulatory region (Upstream+ Downstream)	563,722	8.66	Separate	19.71 ± 1.17	21.65 ± 1.19	33.24 ± 1.29	13.46 ± 1.05
			Joint	3.06 ± 2.31	1.21 ± 2.21	16.52 ± 2.7	3.56 ± 2.06
Exom	55,502	0.8524	Separate	18.33 ± 1.11	19.94 ± 1.13	30.85 ± 1.25	12.09 ± 0.99
			Joint	4.83 ± 2.17	2.68 ± 2.06	1.37 ± 2.35	0.001 ± 1.88

CW, carcass weight; REA, rib-eye area; MS, marbling score; BFT, back fat thickness.

The traits showed considerable differences in the contribution of each function annotation class to genetic variation. For example, variants located in regulatory regions explained 16.52% (46.58%) of the total phenotype (genetic) variation for REA, while this annotation class accounted for only 1.21% (4.89%) of the phenotypic (genetic) variance for MS. The proportion of phenotypic variance of carcass weight explained by the annotation classes was similar, ranging from 3.06% (regulatory region) to 7.78% (intron). However, the differences in contribution of annotation classes were sizable for the other three traits. Intergenic and intron variants were the major contributors to genetic variation for all traits, with their contribution ranging from 22.21% (intron SNPs for REA) to 45.65% (intergenic variants for MS). The contribution of exon variants to phenotypic variance was modest, with values of 4.83%, 2.68%, 1.37%, and 0.0001% for CW, MS, REA, and BFT, respectively. Similarly, regulatory region variants explained 3.06%, 1.21%, 16.52%, and 3.56% of the phenotypic variance for CW, MS, REA, and BFT, respectively.

The annotation subclasses comprising the exon category were also analyzed using both separate and joint computational approaches (Table 4). For all four traits, the estimates obtained from the separate analysis were higher than those from the joint analysis. In addition, the aggregated variances attributed to these subclasses were significantly greater than the total variance explained by the exon annotation class. This discrepancy highlights the overestimation of genetic variance for individual annotation classes when other classes are excluded from the model (separate analysis). There was a considerable variation among annotation subclasses regarding the genetic variance they explained. For example, synonymous variants contributed a negligible (close to zero) proportion of the phenotypic variance for CW, MS, and BFT, while they accounted for 3.58% of the phenotypic variance for REA. The high proportion of variance explained by the exon SNP category for CW primarily originated from missense variants (3.49%). In contrast, other subclasses, such as synonymous and 5′ UTR variants, had a very low contribution. The results also showed that “other regulatory variants” play a significant role in the inheritance of both MS and BFT.

**Table 4:** Explained genetic variance of the studied traits due to different components of the “Exome” category.

**Category of exons**	**No.SNPs**	**%**	**Analysis**	**CW**	**MS**	**REA**	**BFT**
Missense	13,140	0.2018	Joint	3.49 ± 1.43	0.12 ± 1.29	0.26 ± 1.53	0.0001 ± 1.23
			Separate	15.87 ± 1.03	16.8 ± 1.05	26.07 ± 1.18	10.26 ± 0.91
Other regulatory variants	5,480	0.0842	Joint	0.46 ± 1.48	1.07 ± 1.15	0.0001 ± 1.22	0.174 ± 1.03
			Separate	13.26 ± 0.91	14.76 ± 0.95	20.01 ± 1.02	8.85 ± 0.81
Synonymous	22,284	0.3422	Joint	0.0001 ± 1.8	0.0001 ± 1.76	3.58 ± 2.004	0.001 ± 0.1.58
			Separate	17.18 ± 1.07	18.69 ± 1.09	29.03 ± 1.2	11.18 ± 0.94
3′ UTR	11,720	0.18	Joint	0.84 ± 0.91	1.29 ± 0.91	0.0001 ± 0.98	0.0001 ± 0.78
			Separate	13.57 ± 0.97	15.16 ± 1.01	23.32 ± 1.16	7.26 ± 0.78
5′ UTR	2,878	0.0442	Joint	0.0001 ± 0.69	0.19 ± 0.67	0.051 ± 0.72	0.106 ± 0.609
			Separate	8.22 ± 0.74	10.5 ± 0.81	16.31 ± 0.97	6.09 ± 0.66

CW, carcass weight; REA, rib-eye area; MS, marbling score; BFT, back fat thickness.

### 3.4 Genetic variance per SNP

**Figure 9:**
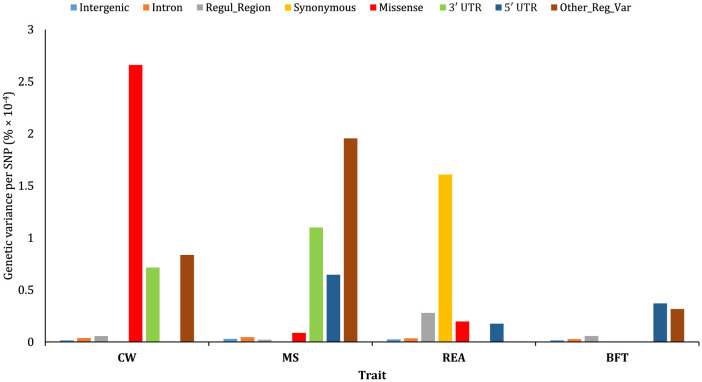
Estimated proportion of genetic variance of the traits studied captured on a per SNP basis for the functional annotations obtained using joint analysis.

The results revealed that the explained genetic variance per SNP for intergenic, intron, and regulatory region categories (except for REA) is notably low (Figure 9). Also, the proportion of variance explained per SNP varied considerably across traits within each annotation class. Synonymous variants had the highest genetic variance explained per SNP for REA, while for BFT, “other regulatory variants” and 5′ UTR SNPs exhibited significantly higher genetic variance per SNP compared to other annotation classes. Interestingly, CW and MS showed a different pattern regarding average genetic variance explained by each SNP. Missense mutation variants had the largest contribution to genetic variance for CW, while their contribution to MS was minimal. Conversely, 5′ UTR variants contributed significantly to the genetic variance of MS but had a negligible impact on CW. Additionally, 3′ UTR annotations played a considerable role in the genetic variation of both CW and MS.

## 4 Discussion

This study deciphered the genomic variance of the carcass weight, marbling score, rib-eye area, and back fat thickness in Angus beef cattle due to several factors using imputed WGS variants. Genotype imputation increased genome resolution, reducing the average SNP distance from 48,397 bp in the Bovine 50K SNP array to 381 bp in imputed WGS. This improvement provided a great opportunity for more accurate partitioning of additive genetic variance by increasing marker density and enabling access to a larger number of low-frequency and causal variants for analysis (3). Consequently, using imputed WGS data is expected to enhance the proportion of genetic variance explained by genotype information. The estimated heritabilities were 0.22, 0.25, 0.35, and 0.15 for CW, MS, REA, and BFT, respectively, reflecting a relatively considerable genetic contribution to the phenotype variation of the traits. These estimates were in the range of previously reported heritability estimates in other cattle populations (29–32). However, higher heritability estimates based on pedigree-based relationships have been reported by Smith et al. (33) and Kause et al. (34). This discrepancy could be attributed to the applied methods, as heritability estimates derived from pedigrees are often higher than those based on genomic relationship matrices (35, 36).

Two methods, GREML and BayesR, have been commonly used for genomic variance partitioning. Recently, Yuan et al. (37) demonstrated that the accuracy of variance component estimation by these models depends on the genetic architecture of the trait. We applied the GREML algorithm implemented in the GCTA program (28) due to its computational efficiency, ability to provide unbiased variance-component estimates (20), and flexibility in partitioning variance attributable to subsets of SNPs in large datasets. In this study, genetic variance of carcass weight and meat quality traits in Angus beef cattle was partitioned into different components based on MAF, chromosomes, and functional annotation. Each component was considered a random effect in a mixed linear model, where genetic covariances among individuals were modeled using a GRM constructed by a subset of SNPs corresponding to the component. The components were fitted either separately in single-random effect models (separate analysis) or simultaneously in a multi-random effect model (joint analysis). The relatively huge data size used in this study (6.5 million SNPs for 13,241 samples) met the requirements of sample size and genotype coverage proposed by Abdollahi-Arpanahi et al. (11) and Zhang et al. (3) for obtaining more reliable estimates. In addition, Ogawa et al. (17) stated that the smaller sample size can complicate the interpretation of results due to spurious LD structure.

The results showed that fitting a subset of SNPs as a single random effect, while ignoring the other SNPs in the model, results in overestimation of the variance components. For all three investigated factors (chromosome, MAF, and functional annotation), the genetic variance estimates obtained from separate analyses were substantially higher than those estimated using joint analysis. Notably, the proportion of genetic variance explained by each MAF bin or functional annotation class relative to the phenotypic variance of the trait was close to, or slightly lower than, the total heritability of the trait. Also, the accumulated genetic variance of the subsets estimated using separate analyses greatly exceeded the genetic variance of the trait estimated using the entire SNPs fitted at once. Similar findings have been reported by Abdollahi-Arpanahi et al. (11) and Bhuiyan et al. (1).

The overestimation of genetic variance components in separate analyses may result from linkage disequilibrium (LD) between the subset of SNPs included in the model and those excluded. These findings support that SNPs will strongly carry over the effects of their neighbors' variants, leading to biased estimates (11, 38). Therefore, the estimates cannot be interpreted as the proportion of variance explained solely by the subset of SNPs. A comparison of the estimated genetic variance due to functional annotation classes and autosomes further illustrates this issue. Different genome regions, such as intergenic and genic regions, including regulatory regions (promoters, enhancers, terminators, etc.), exons, and introns, are tightly linked and are expected to exhibit high LD.

In contrast, there is no physical linkage between variants on different chromosomes. However, they could be involved in the same gene expression network. Abdollahi-Arpanahi et al. (11) stated that selection programs have resulted in negative LD between markers and causative genes in chicken populations, even on different chromosomes.

On the other hand, the total genetic variances (aggregated estimates due to all random effects in the model) were similar to those obtained by fitting all 6.5 million variants as a single random effect. This pattern was consistent across all traits. These findings were in agreement with the results reported by Bhuiyan et al. (1) and Jensen et al. (4). They indicated that fitting multiple random effects modeled by GRMs in mixed linear models would appropriately (unbiasedly) partition the total genetic variance of the complex quantitative traits into the components.

### 4.1 Autosomes

The results indicated that all autosomes contributed to trait variation, though significant differences were observed among the chromosomes regarding the explained genetic variance. While chromosome length was highly correlated with the number of harboring variants (Supplementary Figure S1), there was no strong linear relationship between heritability and chromosome length (*R*^2^ < 0.35), reflecting that some short chromosomes can capture a higher proportion of genetic variance, likely due to the presence of major genes or QTLs on these chromosomes. Similar results have been previously published for production, reproduction, and health-related traits in Holstein cattle (3, 4, 39, 40), carcass traits in Korean Hanwoo beef cattle (1), human height (18), and multiple sclerosis in humans (41). Our findings support the idea that carcass weight and meat quality traits in Black Angus cattle are affected by many loci on all autosomes. However, these effects are not equally distributed across the genome, which aligns with the polygenic inheritance model (42).

In this study, the highest variance for CW and BFT was attributed to BTA20, which is a relatively short chromosome. This chromosome harbors the *growth hormone receptor* (*GHR*) gene, which is involved in muscle development processes by activating intercellular signals that promote growth (43). Additionally, some QTLs and genome regions with significant effects on carcass and body weights have been reported on BTA20 by Casas et al. (44), Li et al. (45, 46), Edea et al. (47), and Hay and Roberts (48). Qin et al. (49) identified a genome region on this chromosome, overlapping between three QTLs, associated with body weight and *GHR* in three cattle breeds. In addition, Saatchi et al. (50) reported a large-effect pleiotropic QTL on BTA20 linked to traits such as birth weight, carcass weight, BFT, mature weight, weaning weight, and yearling weight in Angus, Hereford, Red Angus, and Simmental breeds. Additionally, chromosome 7 explained a great proportion (~ 3 times that of BTA1) of the genetic variation of MS in this population. These findings were in accordance with the results of previous studies reporting the presence of QTLs/genomic regions significantly affecting MS in Japanese Black cattle (51–53), commercial American Angus (54), as well as 10 different cattle breeds (50).

### 4.2 MAF bins

We did not observe a consistent pattern in the explained genetic variance across the MAF bins for the studied traits, which reflects the differences in genetic architectures of carcass weight and meat quality traits in Angus cattle. However, the contribution of MAF classes varied significantly among the traits, with all MAF bins contributing, to some degree, to the genetic variation of the traits. Among the MAF bins, SNPs with 0.27 ≤ MAF < 0.38 explained the highest genetic variance across all traits. In addition, more common variants (27 ≤ MAF ≤ 0.5) collectively accounted for at least 50% of the total genetic variance of the traits. Several studies have reported that the variants with common alleles contribute more to the genetic variance of CW and meat quality traits in Korean Hanwoo beef cattle (1) and CW in Japanese Black cattle (17). The recently reported results for schizophrenia (10) and multiple sclerosis (41) traits in humans also support the findings of the present research.

In contrast, Abdollahi-Arpanahi et al. (11) reported that a great proportion of the genetic variance for body weight, breast muscle, and egg production traits in chickens is explained by rare variants (MAF < 0.2). The inconsistency is likely due to the differences between the species studied and, more importantly, the intense artificial selection programs applied to commercial chicken populations.

The lower frequency alleles (0 ≤ MAF < 0.09) explained a considerable proportion (~ > 13%) of the total genetic variance for the studied traits. To further investigate, we partitioned this bin into two subclasses (0 ≤ MAF < 0.05 and 0.05 ≤ MAF < 0.09). The results revealed that a great portion of the variance in the traits, ranging from 6.23% (MS) to 16.39% (CW), was explained by rare variants (MAF < 0.05) in the population under study (data not shown). This suggests that some causal variants may be located near rare variants or that these variants can relatively model the family relationships within the population (55). Similarly, Zhang et al. (3) reported that SNPs with MAF < 0.05 had a larger contribution to the genetic variance of health-related traits compared to production traits in Holstein cattle. It has also been reported that a considerable proportion of fertility traits in Holstein cattle could be explained by rare variants (56).

### 4.3 Functional annotations

The imputed WGS variants were classified into major functional annotation classes, including intergenic (58.97%), intron (31.52%), regulatory region (8.66%), and exon (0.85%) variants. The distribution of SNPs across annotation classes was close to that reported by Santana et al. (12) in Nellore cattle, where 63.74% and 28.17% of SNPs were intergenic and intronic, respectively. Similarly, Koufariotis et al. (2) reported 67.0%, 31.0%, 8.0%, and 1.0% of HD bovine chip SNPs (777-K) data were intergenic, intron, regulatory region, and exon variants, respectively, in beef cattle. However, Bhuiyan et al. (1) reported that 70.30%, 28.79%, and 0.88% of the imputed WGS SNPs were intergenic, intron, and exon variants, respectively. This inconsistency is probably due to the differences in imputation strategy and the fact that these authors have considered the regulatory variants and intergenic regions as a single class.

However, all functional annotation classes contributed significantly to the traits considered in this study, though notable differences were observed in the distribution patterns of genetic variance explained by these classes. For carcass weight, the estimates were in a relatively small range (14–35.6%), while a wider range was observed for the three other traits. These findings reflect the distinct biological nature of CW compared to the other studied traits in Angus beef cattle. The contribution of genome annotations appears to depend on both the traits and species. Morota et al. (14) studied the body weight, area of breast meat, and egg production traits in chickens using a 600K SNP array and reported a variation in predictive ability of different functional annotations among the traits. These authors recommended using all markers to predict complex traits. In contrast, Do et al. (13) stated that the predictive accuracy across genomic annotations was similar for residual feed intake and its component traits, such as daily feed intake, average daily gain, and back fat. However, these authors utilized only 30,234 SNPs, a relatively small dataset compared to imputed WGS data.

The regulatory region and exon variants showed considerable differences among the traits. For instance, regulatory region variants explained a great proportion of the total genetic variance for rib-eye area (46.58%), while their contribution was < 5% for MS. Interestingly, exon variants showed a broad range of contribution, explaining 22.11%, 10.85%, 3.85%, and 0.00% of the genetic variance for CW, MS, REA, and BFT, respectively, despite comprising only 0.8% of all SNPs. In addition, the annotation subclasses of the exon variant category showed great differences in explained genetic variance, both within and between the traits. The missense SNPs contributed significantly to the genetic variance of CW (3.49%), while other subclasses, in particular synonymous and 5′ UTR variants, had a negligible contribution. The synonymous variants captured a considerable proportion of the phenotypic variance of REA (3.58%), while their contribution was very low (close to zero) for all the other traits. The “other regulatory variants” class, including splice acceptor, splice donor, splice region, start lost, stop lost, and stop retained variants, captured a higher proportion of genetic variance for BFT than the other exon class subclasses. Yang et al. (9) reported that genic regions proportionally explained more variation than intergenic regions, likely due to the physical proximity of causal variants to functional genes. Previous results in dairy and beef cattle (2) were in agreement with our results, emphasizing that higher genetic variances are attributed to genic regions compared to intron and intergenic regions. In contrast, Abdollahi-Arpanahi et al. (11) reported that the largest proportion of genetic variance for production traits in broiler chickens is explained by the synonymous SNPs. In addition, Bhuiyan et al. (1) stated that the synonymous class explained significantly more genomic variances than other functional classes. These inconsistencies may be due to differences in traits, species, breeds, LD patterns, and genotyping platforms.

### 4.4 Genetic variance per SNP

To further investigate, we also calculated the proportion of the genetic variation explained per SNP in each functional annotation class. This value reflects the average genetic variance per SNP when all other annotation classes are simultaneously fitted in a multiple-random-effect model. The results revealed considerable differences in genetic variance explained per SNP, both within and between the traits. Missense, synonymous, “other regulatory variants,” and 5′ UTR variants showed the highest contribution to the genetic variance of CW, REA, MS, and BFT, respectively. These findings suggest that protein-coding region variants are more important for traits associated with muscle development (e.g., CW and ERA), while regulatory-related region variants play a more critical role in traits related to lipid and fat metabolism, such as MS and BFT. Koufariotis et al. (2) reported that protein-coding variants explained most of the genetic variance for dairy traits, while UTR annotation classes were more relevant for fat percentage. Additionally, our findings were quite in agreement with those reported by Bhuiyan et al. (1), who proposed that differences in the genetic variance explained by functional classes among traits are attributable to the distinct genetic architecture underlying biological processes for muscle development and fat biosynthesis.

Intergenic and intron functional annotations showed a great contribution to the genetic variance of the traits. However, the genetic variance explained per SNP in these classes was notably low. These differences are likely due to the extremely high number of SNPs in these classes compared to the others. In this regard, Yang et al. (38) demonstrated that when the genomic relationships are not adjusted for incomplete LD between SNPs and causal variants, the proportion of explained genetic variance increases with the number of SNPs.

## 5 Conclusion

This study applied genomic-based mixed linear models to partition the genetic variation of carcass weight and meat quality traits in Angus beef cattle according to MAF bins, functional annotations, and autosomal chromosomes. Our findings revealed that while most genetic variation is attributable to common alleles, rare variants also explained a significant proportion. Considerable differences were observed among functional annotations both within and between traits, suggesting two key genetic mechanisms underlying these traits in beef cattle: muscle development and lipid biosynthesis. Almost all components (e.g., functional annotations, chromosomes, and MAF bins) contributed to the genetic variation of the studied traits, supporting a polygenic inheritance model. Overall, this study provides comprehensive and valuable insights into the genetic architecture of carcass and meat quality traits, offering opportunities to enhance genomic prediction accuracy and develop more efficient breeding strategies in Angus beef cattle.

## Data Availability

The whole genome sequence data of the samples used as a reference population in this study are available from the European Nucleotide Archive (ENA) under project number PRJEB42783 (https://www.ebi.ac.uk/ena/browser/view/PRJEB42783).
