# Supplementary material for: Genomic variance partitioning of carcass and meat quality traits in Angus beef cattle
Source: Front Vet Sci. 2025 Jun 18;12:1590226. doi: 10.3389/fvets.2025.1590226 (PMC12218258; doi:10.3389/fvets.2025.1590226)
Supplement: Supplementary file 1 [file Data_Sheet_1.pdf]

Genomic variance partitioning of carcass and meat quality traits in Angus beef cattle

Supplementary Materials

1 Supplementary Figures

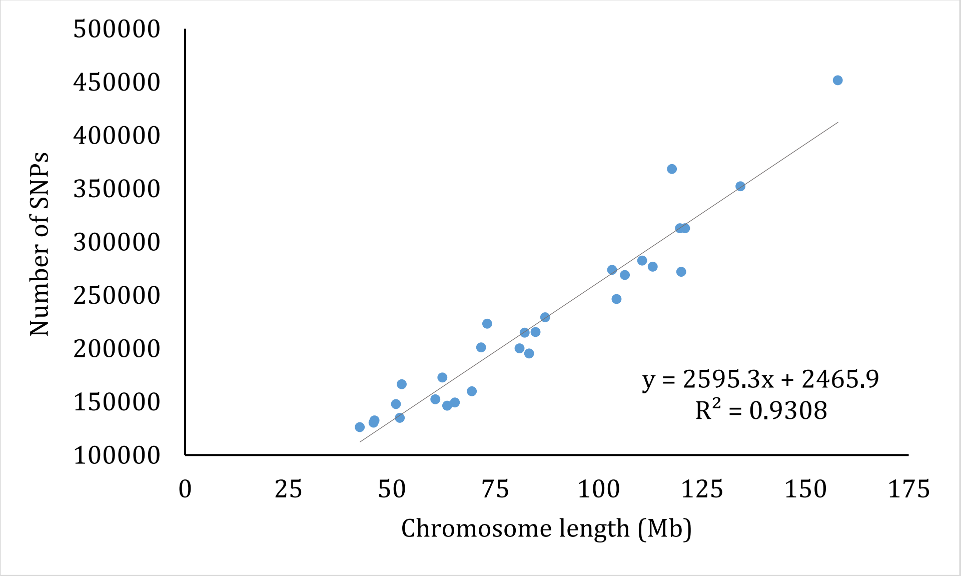

Supplementary Figure S1. Relationship between number of SNPs and physical length of the chromosome

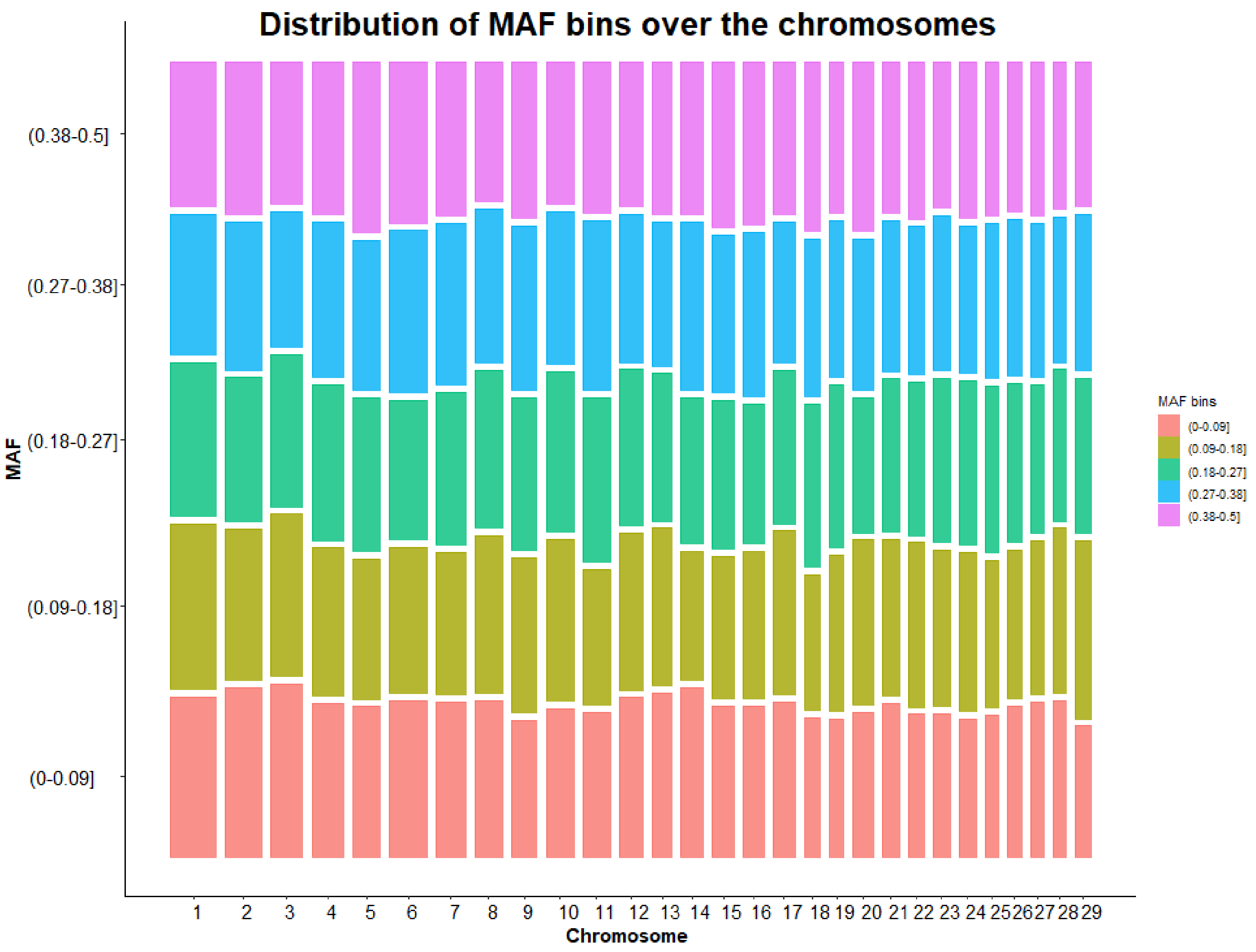

Supplementary Figure S2. Distribution of the MAF bins over the chromosomes.

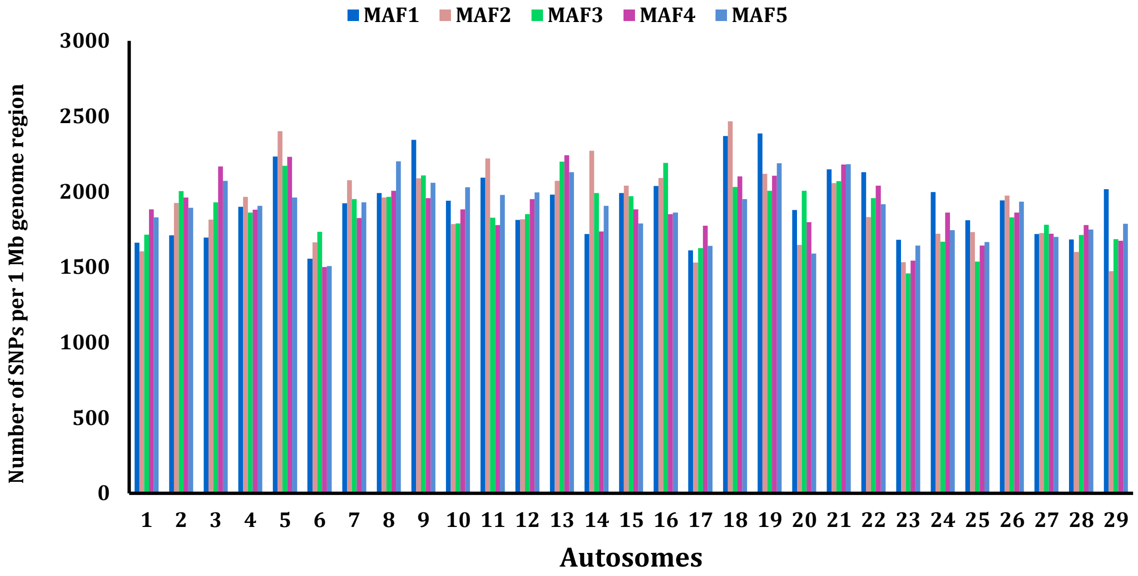

Supplementary Figure S3. Marker density of imputed SNPs in different MAF bins across the autosomes

2 Supplementary Tables

Supplementary Table S1. Distributed the number of SNPs over the functional annotation categories

Functional annotation	No. SNPs	%	Annotation class	No. SNPs	%
Intergenic	3840411	58.97	Intergenic region	3840411	58.98
Intron	2052343	31.52	Intron	2052343	31.52
Downstream	274865	4.22	Regulatory region	563722	8.66
Upstream	288857	4.44			
Synonymous	22284	0.3422	Exon	55502	0.8524
Missense	13140	0.2018			
3′ UTR	11720	0.1800			
5′ UTR	2878	0.0442			
"Other regulatory variants"	5480	0.0842			

Other regulatory variants included splice_acceptor_variant (n=34), splice_donor_variant (n=634), splice_region_variant (n=4651), start_lost (n=34), stop_lost (n=111), and stop_retained_variant (n=16).
